# The (Poly)Pharmacology of Cannabidiol in Neurological and Neuropsychiatric Disorders: Molecular Mechanisms and Targets

**DOI:** 10.3390/ijms22094876

**Published:** 2021-05-05

**Authors:** Rosa Maria Vitale, Fabio Arturo Iannotti, Pietro Amodeo

**Affiliations:** 1 Institute of Biomolecular Chemistry, National Research Council (ICB-CNR), Via Campi Flegrei 34, 80078 Pozzuoli, NA, Italy; fabio.iannotti@icb.cnr.it (F.A.I.); pamodeo@icb.cnr.it (P.A.); 2 Endocannabinoid Research Group (ERG), Institute of Biomolecular Chemistry, National Research Council (ICB-CNR), Via Campi Flegrei 34, 80078 Pozzuoli, NA, Italy

**Keywords:** cannabidiol, pharmacology, neuropsychiatric disorders, receptors, pharmacological targets

## Abstract

Cannabidiol (CBD), the major nonpsychoactive Cannabis constituent, has been proposed for the treatment of a wide panel of neurological and neuropsychiatric disorders, including anxiety, schizophrenia, epilepsy and drug addiction due to the ability of its versatile scaffold to interact with diverse molecular targets that are not restricted to the endocannabinoid system. Albeit the molecular mechanisms responsible for the therapeutic effects of CBD have yet to be fully elucidated, many efforts have been devoted in the last decades to shed light on its complex pharmacological profile. In particular, an ever-increasing number of molecular targets linked to those disorders have been identified for this phytocannabinoid, along with the modulatory effects of CBD on their cascade signaling. In this view, here we will try to provide a comprehensive and up-to-date overview of the molecular basis underlying the therapeutic effects of CBD involved in the treatment of neurological and neuropsychiatric disorders.

## 1. Cannabidiol

**Figure 1:**
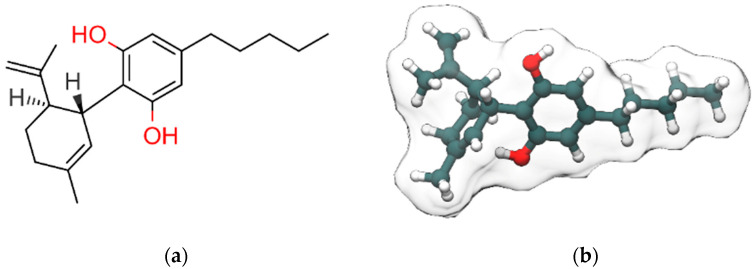
2D (**a**) and 3D (**b**) representations of cannabidiol (CBD). Hydroxyl group (2D view)/oxygen atoms, carbon atoms and hydrogen atoms (3D view) are colored red, dark slate gray and white, respectively. Only polar and stereo hydrogen atoms (the latter colored in gray) are shown in (**a**).

Cannabidiol (CBD, Figure 1), along with Δ^9^-tetrahydrocannabinol (Δ^9^-THC), is the most abundant bioactive compound of *Cannabis sativa*. Differently from Δ^9^-THC, it is devoid of any psychotropic effects [1]. Interestingly, Δ^9^-THC and CBD are often considered the yin and the yang of cannabis extract for their antithetic effects: Δ^9^-THC binds with high affinity and activates cannabinoids receptors, responsible for the rewarding effects of cannabis, while CBD has a low affinity for the orthosteric sites of those receptors and acts as negative allosteric modulator (NAM) at Cannabinoid receptor 1 (CB1R) in the nanomolar range [2]. The NAM effect of CBD at CB1R was confirmed in a recent study by Tham et al. [3] while at CB2R it behaves as partial agonist. Moreover, CBD counteracts the anxiogenic effects of Δ^9^-THC and, due to its effects in inhibiting drug relapse, is currently under investigation for the treatment of addiction disorders [4]. CBD, whose structure was elucidated by Mechoulam and Shvo [5] in 1963, has been recently approved by the US Food and Drug Administration (FDA) and the European Medicines Agency (EMA) as an antiepileptic drug (Epidiolex) for the treatment of patients affected by refractory epilepsy such as Dravet [6] and Lennox–Gastaut syndromes [7]. Here, we discuss the therapeutic potential of CBD in neurological and neuropsychiatric disorders with particular emphasis on the involved molecular targets and mechanisms. The review is organized in two main sections: the first one reports an overview of the pharmacological effects of CBD in neurological and neuropsychiatric disorders, the second one describes the molecular targets and the molecular mechanisms involved in these effects. Structural details from experimental structures of the ligand-binding sites are also discussed, along with mutagenesis data.

## 2. Neurological and Neuropsychiatric Disorders Potentially Affected by CBD Treatment

### 2.1. Epilepsy and Cannabidiol

Epilepsy is a very frequent group of neurological disorders affecting around 50 million people worldwide. It is caused by excessive and abnormal brain activity, characterized by recurrent seizures and neuropsychiatric comorbidities, which negatively affect the quality of life of patients. Despite the multiple antiepileptic drugs (AEDs) available, about one-third of adults and approximately 20–25% of children have forms of epilepsy that do not respond to drug therapy [8] and frequently these patients receive higher doses of multi-AED regimens that are not only ineffective but also cause unpleasant side effects. For this reason, there is still an urgent need to find treatments against drug-resistant epilepsy (DRE) [9,10]. In this view, CBD has received great scientific interest due to its anticonvulsant proprieties, as revealed in experimental animal models of epilepsy [11]. Notably, CBD (Epidiolex), recently reviewed by Abu-Sawwa and Park [12], has been the first cannabis-derived medication approved in 2018 by the FDA for patients ≥2 years of age diagnosed with rare forms of AEDs-refractory epilepsy such as Dravet syndrome (DS) and Lennox-Gastaut syndrome (LGS). The molecular targets possibly involved in mediating the therapeutical effects of CBD in epilepsy include γ-aminobutyric acid (GABA) A receptors (GABA_A_Rs, Section 3.1.1), glycine receptors (GlyRs, Section 3.1.2), transient receptor potential cation channel subfamily V member 1 (TRPV1, Section 3.2.1), transient receptor potential ankyrin subtype 1 protein (TRPA1) and possibly TRPV2 (Section 3.2.2), and GPR55 (Section 3.3.4).

### 2.2. Alzheimer’s Disease and Cannabidiol

Alzheimer’s disease (AD) is a neurodegenerative disorder associated with progressive memory and cognitive impairment, which severely compromises the ability to carry out everyday tasks. It represents the most common cause of dementia, accounting for 60–70% of cases (World Health Organization, 2020). The two pathological hallmarks of AD are the deposition of β-amyloid (βA) peptide, leading to senile plaques, and the hyperphosphorylation of tau protein, forming neurofibrillary tangles (NFTs). Microglia is activated by βA, but its impaired clearance results in the release of inflammatory cytokines and reactive oxygen and nitrogen species, triggering neuroinflammatory processes, neurotoxicity and oxidative damage. Additionally, neurodegeneration increases the levels of glutamate and decreases the cholinergic tone in brain areas involved in memory functions [13,14]. Despite the ever-increasing understanding of the molecular basis of AD, the current approved treatments, such as acetylcholine inhibitors and N-methyl-D-aspartate (NMDA) receptor antagonists, only provide limited symptomatic reliefs. In this view, CBD could represent a promising therapeutic candidate for AD, due to its neuroprotective, anti-oxidant and anti-inflammatory effects [15,16]. Moreover, CBD has been found effective in reducing βA production and tau hyperphosphorylation in vitro [17]. It also exhibits neuroprotection against βA-mediated toxicity and inhibits microglial-activated neurotoxicity [18]. The effects of CBD in in vivo AD models have been recently reviewed by Watt and Karl [13]. The possible role of Peroxisome proliferator-activated receptor, isoform γ (PPARγ, Section 3.5) in mediating the therapeutic effects of CBD in AD models emerges from the studies of Esposito [19] and Scuderi [20]. Other targets involved in mediating CBD effects are 5-hydroxytryptamine (5-HT) type 3 receptors (5-HT_3_Rs, Section 3.1.3).

### 2.3. Parkinson’s Disease and Cannabidiol

Parkinson’s disease (PD) is a neurodegenerative, age-related disorder characterized by both motor and non-motor symptoms. PD patients experience bradykinesia, resting tremor, rigidity, and postural instability. The non-motor symptoms include sleep disturbance, cognitive deficits and psychiatric disorders such as psychosis, depression, and anxiety. PD is a multifactorial disease associated with both genetic and environmental risk factors [21]. The pathological hallmarks of PD are the loss of dopaminergic neurons in the *substantia nigra* and the development of Lewy Bodies in dopaminergic neurons [22]. The pharmacological treatment of PD essentially involves the administration of dopamine precursors (levodopa) and inhibitors of dopamine metabolism (monoamine oxidase (MAO) inhibitors, catechol-O-methyl transferase (COMT) inhibitors). Interestingly, CBD was shown to exert neuroprotective effects in PD animal models, probably mediated by its antioxidant and anti-inflammatory properties [23], and in three trials it was proven to be effective in counteracting non-motor symptoms of PD [24]. CBD molecular targets involved in mediating its therapeutic effects in PD are 5-HT_3_Rs (Section 3.1.3) and GPR6 (Section 3.3.3).

### 2.4. Depression and Cannabidiol

Depression is a widespread, disabling chronic psychiatric disorder characterized by sadness, anxiety, loss of interest and pleasure, seriously affecting a person’s ability to handle daily activities, such as sleeping, eating, or working. Depression can also occur as a comorbid psychiatric condition in chronic diseases. The antidepressant- and anxiolytic-like effects of CBD in animal models have been reviewed by de Mello Schier et al. [25]. In particular, in a comparative study using imipramine as a reference compound, Réus et al. [26] investigated the behavioral and molecular effects of CBD induced by acute and chronic administration in rats. Using the forced swimming test, both imipramine and CBD at 30 mg/Kg reduced immobility time and increased swimming time. Moreover, CBD at 15 mg/Kg and imipramine at 30 mg/Kg increased the levels of the brain-derived neurotrophic factor (BDNF) protein in the rat amygdala. BDNF is a biomarker for depression, since a decrease in the BDNF levels has been observed in both animal models and patients affected by depression. Conversely, the treatment with antidepressants increases BDNF expression and the infusion of BDNF in rat brains produces antidepressant-like effects [27]. BDNF is linked to serotoninergic transmission by promoting development and function of serotoninergic neurons. Furthermore, BDNF infusion into the brain enhances the expression of tryptophan hydroxylase, the rate-limiting enzyme in 5-HT synthesis [28], and potentiates 5-HT release [29]. In turn, 5-HT upregulates BDNF expression levels [30]. Interestingly, Di Giacomo et al. [31] showed that CBD is able to restore the cortex level of 5-HT depleted by neurotoxic stimuli. The molecular targets mainly involved in mediating the CBD antidepressive effects are the 5-HT receptors 5-HT_3A_R (Section 3.1.3) and 5-HT_1A_R (Section 3.3.2), and the two closely related G-protein coupled receptors (GPCRs) GPR3 and GPR6 (Section 3.3.3).

### 2.5. Anxiety Disorders and Cannabidiol

While anxiety is a physiological adaptive response to stress, in the case of anxiety disorders the feeling of fear or apprehension is prolonged, excessive, irrational and debilitating, thus negatively affecting daily life activities. Anxiety disorders represent the most common form of emotional disorders according to epidemiological studies. They include panic disorder, phobia, separation anxiety disorders, illness anxiety disorder, post-traumatic stress disorder, social anxiety disorder and obsessive–compulsive disorder. The multifactorial etiology includes genetic, neurobiological and psychosocial factors [32]. Anxiety disorders frequently occur in patients with chronic medical disorders, further increasing their disability. In the last years, CBD has gained considerable attention due to its anxiolytic effects, recently reviewed by Blessing et al. [33]. In a recent paper, Shannon et al. [34] described the results of a clinical application of CBD among patients affected by anxiety and/or sleep disturbances. Within the limits of the study due to the open-label treatment, absence of a comparison group and the use of concurrent psychiatric medications as part of clinical care routine, the results showed that, while the sleep scores only displayed a mild improvement, anxiety scores decreased in a rapid and sustained manner during the study period. The same CBD targets described for depression, 5-HT_3A_R (Section 3.1.3), 5-HT_1A_R (Section 3.3.2), GPR3 and GPR6 (Section 3.3.3) could be involved in CBD effects on anxiety disorders, together with GABA_A_Rs (Section 3.1.1) and PPARγ (Section 3.5).

### 2.6. Drug Addiction and Cannabidiol

Drug addiction is a chronic, relapsing disorder characterized by drug seeking and compulsive and uncontrolled use, despite the harmful consequences. Drug addiction can begin as the deliberate use of recreational drugs in social contexts, or as exposure to prescribed medications, such as opioid drugs, but in both cases, the repeated use affects the reward circuit causing intense craving and withdrawal symptoms. Addictive disorders are associated with a complex illness, characterized by different stages: in the acute or intoxication phase, the use of drugs alters the brain levels of various neurotransmitters, which leads to the classical “high” effects such as euphoria, restlessness, and tachycardia. Instead, the abstinence phase is characterized by anxiety, dysphoria, sleep disturbance and low tolerance to stress, resulting in recurrent craving and relapse [4]. Since CBD exerts many of its therapeutic effects within the neural circuits involved in the acquisition of addiction and drug-seeking behaviors, it represents a promising pharmacological candidate for the treatment of substance abuse disorders [4,35,36,37]. CBD molecular targets mainly involved in these therapeutic effects are 5-HT_3A_R (Section 3.1.3) and PPARγ (Section 3.5). 

### 2.7. Autism Spectrum Disorder and Cannabidiol

Autism spectrum disorder (ASD) is a complex neurodevelopmental condition characterized by persistent deficits in social interaction, verbal and nonverbal communication, and restricted/repetitive behaviors, interests or activities [38], often associated with a poor quality of life and lack of independence. ASD has a multifactorial etiology with a high genetic component, albeit the expressivity of the disorder is largely influenced by environmental factors [39]. Risk factors include advanced maternal and/or paternal age, maternal metabolic conditions such as obesity, hypertension and diabetes [40]. Alterations of the endocannabinoid system have been implicated in several neuropsychiatric disorders, including ASD, as emerges from studies on animal models of ASD-like behavior [41]. In particular, Karhson et al. [42] suggested that an impaired signaling of the endocannabinoid anandamide is involved in the physiopathology of ASD since children with lower plasma anandamide levels were more likely to have ASD. ASD patients have comorbid epilepsy [43], and several neuronal pathways seem to be involved in both diseases [44]. Due to its therapeutic effects in the treatment of refractory epilepsy, CBD recently emerged as a therapeutic candidate also for ASD symptoms, supporting the feasibility of this treatment for clinical trials in children with ASD [45]. In a recent study [46], the effects of CBD-enriched extract of *Cannabis sativa* (CBD/ Δ^9^-THC ratio 75:1) were evaluated on ASD symptoms of a cohort of 18 autistic patients, indicating that such extract is effective even in non-epileptic patients. Given the emerging role of anandamide in ASD, it is plausible that CBD could exert at least in part its therapeutic action through the inhibition of the anandamide metabolizing enzyme fatty acid amide hydrolase (FAAH) [47]. Other CBD molecular targets involved in ASD are GlyRs (Section 3.1.2).

### 2.8. Psychotic Disorders and Cannabidiol

Psychotic disorders, such as schizophrenia and bipolar disorders, are mental illnesses characterized by an impaired reality testing and symptoms such as false beliefs, (delusions), false perceptions (hallucinations) and incoherent speech. Psychoses can be classified in three main groups: idiopathic psychoses, psychoses due to pathologic conditions (including fevers, epilepsy and neurodegenerative disorders), and toxic psychoses (due to drug abuse or toxins) [48]. Many studies suggest that psychotic symptoms are caused by an alteration in the inhibitory/excitatory circuits due to increased synaptic levels of dopamine and glutamate and a deficiency of GABA and NMDA receptors [48]. The use of cannabis extracts with high levels of Δ^9^-THC, as well the use of synthetic CB1R agonists, have been associated with an increased risk of psychosis and to develop schizophrenia in susceptible subjects, whereas the use of cannabis with higher levels of CBD drastically reduces the probabilities of psychotic experiences [49,50]. The psychotic effects of Δ^9^-THC are due to the activation of CB1 receptor that in turn acts as negative regulator of NMDA receptor (NMDAR), causing its hypofunction [51]. Such NMDAR hypofunction is linked to the dopaminergic dysregulation observed in schizophrenic patients, giving rise to the hypothesis that glutamatergic/NMDA dysfunction underlies the schizophrenia symptoms [52]. Indeed, many studies have demonstrated a physical association between CB1R and NMDAR, occurring through a direct interaction between the C-terminus of CB1R and the C1 segment of NMDAR NR1 subunit [51]. On the contrary, CBD acts as negative allosteric modulator at CB1R [2], which could explain, at least in part, its balancing effects toward the psychotic effects of Δ^9^-THC. Consistently with the involvement of NMDAR, Sigma-1 receptor (σ1R, Section 3.4), could represent another CBD target involved in the observed effects of this ligand in psychotic disorders, which have also been associated with PPARγ (Section 3.5) modulation by CBD.

## 3. CBD Molecular Targets and Mechanisms Involved in the Neurological and Neuropsychiatric Disorders

### 3.1. Cys-Loop Superfamily of Ligand-Gated Ion Channels

#### 3.1.1. GABA_A_Rs

**Figure 2:**
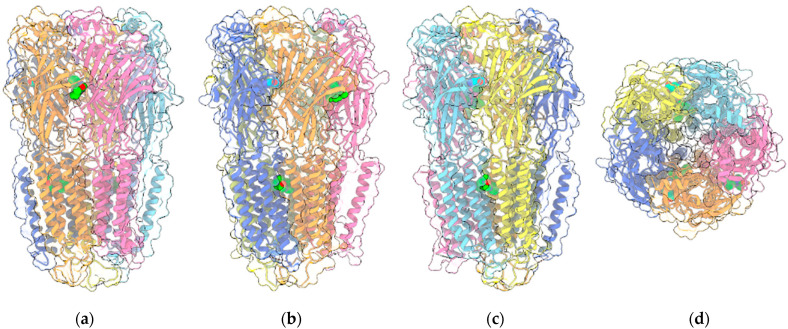
Four views of the 6HUP PDB entry corresponding to the complex of an α_2_β_2_γ GABA_A_R (ribbon plus transparent surface) with three molecules of diazepam (“sphere” representation with green C atoms) and two molecules of GABA (“sphere” representation with cyan C atoms). The two α1, two β3 and the single γ2L monomers are painted light/medium blue, yellow/orange and pink, respectively. Oxygen and nitrogen ligand atoms are painted red and blue, respectively. (**a**–**c**) are perpendicular to the channel axis and aligned either with the α1-γ2L interface (**a**), or the two β3-α1 interfaces (**b**,**c**). (**d**) shows the view along the channel axis. Other ligands included in the entry are omitted.

GABA is the major inhibitory neurotransmitter in the central nervous system (CNS), acting through its cognate receptors GABA_A_Rs and GABA_B_Rs. While GABA_B_Rs are metabotropic G protein-coupled receptors (GPCR), GABA_A_Rs are chloride-selective ion channels, belonging to the Cys-loop superfamily of ligand-gated ion channels, which include nicotinic acetylcholine receptors, 5-HT_3_Rs and GlyRs (Figure 2).

GABA_A_Rs, as other Cys-loop members, feature a pentameric structure composed of different subunit types (α1–6, β1–4, γ1–3, δ, ε, π, and θ) arranged in a α_2_β_2_γ stoichiometry around a central membrane-spanning pore. Albeit this heterogeneity is even amplified by the occurrence of splice variants for each subunit [53,54], the major synaptic isoform involves α1β2γ2 subunits. The subunits have a common topology, consisting of a large extracellular N-terminal domain (ECD) containing the Cys-loop, the signature motif of this class of receptors, where two cysteine residues separated by 13 residues form a disulfide bond, followed by four transmembrane helices M1-M4 (transmembrane domain, TMD) and by the extracellular C-terminus. M2 lines the ion channel, while M3 and M4 helices are linked by a large intracellular loop, which is an interaction site for proteins involved in the modulation of the channel [54]. Dysfunctions of GABA_A_Rs are associated with neurological and psychiatric disorders including epilepsy, insomnia, anxiety, panic and schizophrenia [55,56]. Preclinical studies with transgenic mice and/or selective ligands suggested that diverse receptor isoforms have a different role in mediating the action of drugs in specific disorders: α1 is implicated in epilepsy and in sedative/hypnotic effects, α2/α3 mediate anxiolytic and analgesic actions, α5 is involved in learning and memory, β1 in sleep and β3 in anesthesia [57,58]. GABA_A_Rs represent a target for a large array of drugs such as benzodiazepines (BDZ), barbiturates, anesthetics and neurosteroids, which act as allosteric modulators at distinct binding sites of this receptor channel [59]. Structures of human α1β2γ2 and α1β3γ2L GABA_A_Rs have been recently solved by cryo-electron microscopy [60,61,62] in complex with diverse modulators: the α1β2γ2 isoform with GABA, the competitive antagonist at the benzodiazepine binding site flumazenil, and the anesthetic phenobarbital, etomidate and propofol, while α1β3γ2L isoform with GABA, the channel-blocker picrotoxin, the competitive antagonist bicuculline, and alprazolam and diazepam (DZP) benzodiazepines. The binding site for GABA is located at the βα ECD interfaces, while the benzodiazepines site is at the αγ ECD interface. Phenobarbital binds at two sites in the TMD: the βα and γβ interfaces, in pockets formed in both cases by M1-M3 segments, while etomidate and propofol only bind at the βα interfaces, in a pocket overlapping that of phenobarbital [60]. Besides the classical benzodiazepine binding site, diazepam also binds in βα TMD interface, in a position equivalent to etomidate and propofol, and this additional site is responsible for DZP anesthetic effect and biphasic GABA_A_R potentiation at higher concentrations [62]. Sigel et al. [63], in an elegant study, provided evidence that the endocannabinoid 2-arachidonoyl glycerol (2-AG) is an endogenous allosteric activator of α1β2γ2 GABA_A_R potentiating currents elicited by GABA 1 μM in a concentration-dependent manner. These authors showed that, while the replacement of α1 subunit with α2-6 has little effect on 2-AG potentiation, it is abolished in receptors containing the β1 subunit and strongly reduced by the replacement with β3. Moreover, the potentiation of 2-AG is also reduced when the γ2 subunit is omitted. To identify the binding site of 2-AG, its effect was evaluated in α1β2γ2 receptors bearing the β2(N265S) mutation, which abolishes the potentiation by loreclezole. However, the effect of 2-AG in this mutant is only partially reduced, suggesting a different binding site for 2-AG. To identify the residues involved in 2-AG selectivity, the residues not-conserved between β2 and β1/3, i.e., β2(M294), β2(L301) in M3 and β2(V436) and β2(F439) in M4, were mutated into the respective residues present in β1/3. All the mutations significantly reduced the potentiation by 2-AG and in α1β2(V436T)γ2 it was abolished, suggesting that the effect of 2-AG is mainly mediated by V436 and F439 residues, located on the same face of M4 helix. Moreover, 2-AG acts at this receptor isoform in a superadditive manner with neurosteroids and diazepam and it is able to modulate α1β2δ receptors, in which γ2 is replaced by δ subunit. Bakas et al. [64] identified CBD as a positive allosteric modulator of GABA_A_Rs regardless of α subunit compositions, albeit it exhibits higher efficacy at receptors containing the α2 subunit. The study has been also carried out in parallel on 2-AG. The authors found that both CBD and 2-AG exhibit higher efficacy at α2β2γ2L and α2β3γ2L isoforms than at the α2β1γ2L one, thus showing a preference for β2/β3 over β1 subunit. However, while CBD is significantly more potent at α2β3γ2 than either at α2β1γ2 or α2β2γ2 (EC_50_ 4.4 μM vs. 17.4 ad 16.1 μM, respectively), 2-AG exhibited a comparable EC_50_ at the three isoforms. This result differs from that reported by Sigel and coll. [63], who found a lower modulatory effect at receptor isoforms containing β1 and β3 subunits in comparison to β2, probably due to the use of a different α subunit in the two studies. The β2(V436T) mutation affects the GABA potentiation effect of both CBD and 2-AG, albeit the potencies are similar to the wild-type receptor. CBD (10 μM) and 2-AG (10 μM) were shown to significantly left-shift the GABA concentration-response curve without affecting maximal GABA current. Moreover, low GABA concentrations, below its EC_50_, are significantly enhanced by these agents, whereas a lower potentiation effect occurs at concentrations above EC_50_, similarly to BDZ. The same authors found that the action of CBD is not mediated by the benzodiazepine α2γ2L interface since it is also active at the binary receptor isoforms. Moreover, the potency of CBD at α2β2 was significantly higher than at the ternary α2β2γ2L complex (EC_50_ 2.0 vs. 6.5 μM). As previously found by Sigel et al. for 2-AG [63], both compounds also enhance the extrasynaptic GABA_A_Rs containing the δ subunit. CBD shows greater enhancement than 2-AG (752 % vs. 480% at α4β2δ, respectively) but 2-AG was 5-fold more potent than CBD (EC_50_ 4.8 μM vs. 23.1 μM, respectively). The activity of CBD at GABA_A_Rs containing α2 subunit could account for the anti-seizure, anxiolytic and analgesic effects of this compound found in many preclinical studies [1,65,66].

#### 3.1.2. GlyRs

GlyRs, as other members of Cys-loop superfamily, are pentameric ligand-gated ion channels, which enable the influx of chloride ions. Functional GlyRs arise from different combinations of their four α subunit isoforms α1–4 and the single subunit isoform β. GlyRs mainly occur as heteromers formed by two α and three β subunits [65], anchored to the post-synaptic membranes through the protein gephyrin [66]. The α subunits are characterized by a different temporal distribution: while α2 and α4 subunits are involved in neuronal development, being mainly expressed in embryonic CNS, α1 and α3 mediate the majority of glycinergic inhibitory neurotransmission in the adult spinal cord and brain stem [67,68]. Additionally, α3 is expressed in the hippocampus where it is implicated in temporal lobe epilepsy [69]. Alterations of GlyRs functionality have been associated with autism spectrum disorder (ASD) [68]. CBD was shown to potentiate glycine currents in HEK293 cells expressing α1 and α3 subunits [70,71]. NMR studies carried out on purified α3 GlyRs allowed the identification of the binding site of CBD, unveiling a direct interaction between CBD and S296 residue, located on the third transmembrane domain [71]. The crucial role of S267 in the transmembrane region of α1 subunit in mediating the glycine-enhancing effect of CBD was shown by Foadi and co-workers [72] since the modulatory effect of CBD is lost in HEK293 cells expressing the homomeric mutated form of the receptor α1(S267I).

#### 3.1.3. 5-HT_3_Rs

5-HT_3_Rs are the only serotonin (5-hydroxytryptamine, 5-HT) receptors belonging to the Cys-loop family of ligand-gated ion channels, since the other 5-HTRs are coupled to G proteins [73]. They mediate the fast excitatory neurotransmission of serotonin. Besides the well-known effect of 5-HT_3_R antagonists in the prevention of nausea and vomiting in chemotherapy, many studies have suggested a potential role in neurodegenerative and neuropsychiatric disorders including seizure, memory disorders, eating disorders schizophrenia, depression, anxiety, and drug addiction, as recently reviewed by Fakhfouri and coll. [73]. 5-HT_3_Rs, as other members of the Cys-loop family, are composed of five subunits arranged around a central membrane-spanning pore permeable to sodium, potassium and calcium ions. So far, five 5-HT_3_R subunits (A-E) have been identified, even though those most extensively characterized are 5-HT_3A_R and 5-HT_3B_R. 5-HT_3_Rs are expressed both in the central and in the peripheral nervous system. Activation of presynaptic 5-HT_3_Rs induces a rapid influx of calcium, causing a release of neurotransmitters and neuropeptides, while postsynaptic 5-HT_3_Rs are associated with fast excitatory sodium and potassium-dependent depolarization. Due to the involvement of 5-HT_3_Rs in processes related to cognition and emotion, the use of 5-HT_3_R antagonists has been proven to be beneficial in the treatment of various psychiatric disorders [74], as recently reviewed by Juza et al. [75]. CBD was found to act as an allosteric inhibitor at 5-HT_3A_R expressed in *Xenopus laevis* oocytes, inhibiting the currents evoked by 1μM 5-HT in a concentration-dependent manner with an IC_50_ = 0.6 μM [76].

### 3.2. TRP Channels

#### 3.2.1. TRPV1

**Figure 3:**
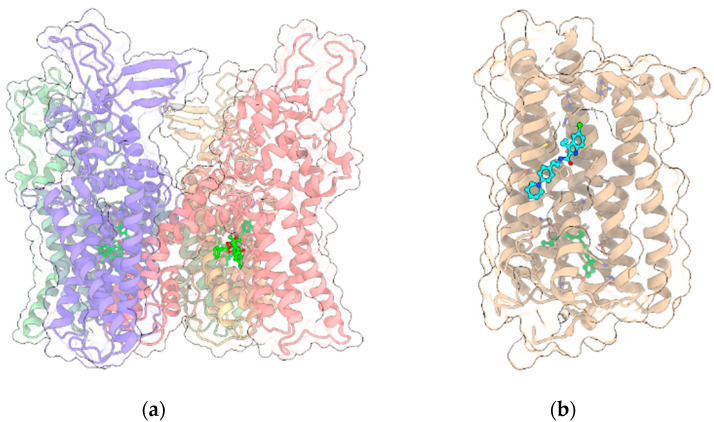
(**a**) Crystal structure (PDB entry: 5IRX) of the 4:4 complex between rat TRPV1 (light green, red, yellow and violet ribbon and transparent surface for the four protein monomers) and the agonist resiniferatoxin (ball and stick with bright green C atoms); (**b**) Crystal structure (PDB entry: 6KQI) of the complex of CB1R (tan ribbon and transparent surface) with the negative allosteric modulator ORG27569 (ball and stick with cyan C atoms) and the agonist CP55940 (ball and stick with bright green C atoms). In all panels, other ligands and non-receptor protein sequences are omitted; protein sidechains contacting ligands within 5 Å are shown as sticks; oxygen, nitrogen, sulfur and chlorine atoms in ligands and visible protein sidechains are colored red, blue, yellow and green, respectively.

TRPV1 was the first member of the large family of TRP channels (Figure 3a) to be discovered and cloned [77]. All TRPV members (TRPV1-TRPV6) are homotetramers sharing a similar architecture, with the transmembrane region of each monomer arranged in a voltage-sensor-like domain (VSLD) and forming the pore channel, while the cytosolic N-terminus contains ankyrin repeat domains (ARDs). The three-dimensional structures of TRPV1 in both apo-form and complexed to agonist/antagonists have been recently elucidated by cryo-electron microscopy [78,79,80]. As with other members of the family, TRPV1 is expressed in various types of both excitable and non-excitable cells and, upon activation, behaves as a non-selective cation channel, conducting divalent as well as monovalent cations, with a preference for the formers and, in particular, Ca^2+^, showing the overall permeability order: Ca^2+^ > Mg^2+^ > Na^+^ ≈ K^+^ [77]. The channel is activated by various stimuli including exogenous vanilloids such as capsaicin, endogenous lipid molecules such as endocannabinoids, eicosanoids, temperature and/or low pH [81]. The binding site of capsaicin and other TRPV1 modulators is located in the so-called vanilloid pocket, formed by the transmembrane helices S3, S4, the S4–S5 linker of one monomer and S6 helix of the adjacent monomer. The importance of TRPV1 channels in the nervous system has been well documented. In particular, TRPV1 expression is restricted to specific brain regions including the hippocampus and cortex with additional expression in the hypothalamus, olfactory nuclei, dentate gyrus, locus coeruleus, superior colliculus and spinal cord [43,44], where it plays a critical role in regulating the cell excitability, long-term depression (LTD), synaptic plasticity and reactive oxygen species production triggering cell death [82,83,84,85].

A massive amount of evidence has demonstrated that hyperactivation/overexpression of TRPV1 contributes to epilepsy and neuroinflammation-induced seizures. In 2010, Bhaskaran and Smith conducted a pioneering study demonstrating that TRPV1 expression was significantly higher in the dentate gyrus of mice with Temporal lobe epilepsy (TLE) compared with control mice and also that anandamide, an endocannabinoid endowed with an intrinsic agonist activity on TRPV1, enhanced glutamate release [86]. Subsequent studies have demonstrated that TRPV1 knockout mice show a decrease in susceptibility to generalized clonic seizures induced by pentylenetetrazol (PTZ) [87], as well as reduced expression of pro-inflammatory markers (IL-1β, IL-6, TNF, and HMGB1) [88]. Similar results have been obtained with TRPV1 antagonists by other research groups [89]. Furthermore, changes in TRPV1 expression and/or activity are recognized as major contributors to the etiology of epilepsy in humans [90]. One of the key mechanisms that modifies channel activity and thus alters the intrinsic electrophysiological properties of TRPV1 is the change in its phosphorylation/dephosphorylation state. In particular, phosphorylation of TRPV1 by intracellular protein kinases such as PKC, PKA and CaM-Kinase II increases channel sensitivity to both chemical and thermal stimuli, whereas receptor dephosphorylation by phosphatases (e.g., calcineurin) causes its inactivation [91]. Intriguingly, Iannotti et al. [92] demonstrated that TRPV1 was strongly phosphorylated and hence over-activated, in the hippocampus of epileptic rats. CBD, along with its analog cannabidivarin (CBDV), were shown to dose-dependently activate and rapidly desensitize TRPV1 both in vitro and in vivo, strongly supporting the hypothesis that the antiepileptic mechanism of CBD and CBDV occurs, at least in part, via TRPV1 desensitization.

#### 3.2.2. Other TRP Channels

Iannotti et al. [92] demonstrated that, besides TRPV1, CBD and CBDV activate and rapidly desensitize other TRP channels including TRPV2 and TRPA1. While this study represents, to the best of our knowledge, the first report where TRPV2 has been directly associated with models of epileptiform activity and acute seizure, in a recent study Günaydın et al. [93] showed that the long term activation of TRPA1 channels by its agonist trans-cinnamaldehyde (TCA) causes an exacerbated PTZ-induced seizure activity in rats. Based on these pieces of evidence, although drugs interacting with multiple targets have long been flagged as undesirable, the story of CBD as an antiepileptic drug has so far demonstrated that molecules hitting more than one target may show not only a higher efficacy compared to the canonical single-target AED drugs, but also a better safety profile [94,95].

### 3.3. GPCRs

#### 3.3.1. CB1R

As stated before, the psychotropic effects of Δ^9^-THC and the synthetic cannabinoids are due to the activation of the cannabinoid receptor CB1R, one of the most highly expressed GPCRs (Figure 3b) in the CNS, where it mediates the signaling of the endocannabinoids N-arachidonoylethanolamine (AEA) and 2-AG. CB1R is also widely expressed in the peripheral nervous system and it primarily couples to G_i/o_ protein, inhibiting the production of cAMP and adenylyl cyclase. However, in certain conditions, CB1R can also couple to G_s_ and G_q_ proteins [96]. CB1R regulates the activity of various ion channels and modulates the release of neurotransmitters by inhibiting the synaptic release of glutamate and GABA, the latter through inhibition of N-type voltage-gated calcium channels [97,98,99]. The reduction of glutamate release due to CB1R activation in presynapses contributes to NMDAR hypofunction. Moreover, NMDA receptors are also negatively regulated by CB1R in postsynapses [100]. Indeed, the C-terminus of CB1R associates with the C1 segment of NMDAR NR1 subunit and the protein HINT1 stabilizes this complex. The activation of CB1R results in a reduction of the NMDAR activity. Due to its wide distribution in the nervous system, CB1R is involved in various physio-pathological processes and is considered a relevant molecular target for the pharmacological treatment of pain, inflammation, multiple sclerosis, nausea, obesity and substance abuse disorders. For example, the Δ^9^-THC/CBD association is the active principle of sativex, approved for the treatment of spasticity, nausea and pain [101]. Recently, the crystallographic structures of CB1R in complex with either agonists or antagonists and negative allosteric modulators have been solved [102,103,104], shedding light on the molecular mechanisms of activation/regulation. While the orthosteric binding site is located in a pocket formed by the transmembrane helices near the N-terminus, the allosteric binding site for the negative allosteric modulator (NAM) ORG27569 is extrahelical, toward the C-terminus, and overlaps a conserved site for cholesterol. The studies of Laprairie et al. [2] showed that CBD behaves as NAM at CB1R and such activity, as for ORG27569, is influenced by the occurrence of polar residues at positions 98 and 107, corresponding to two cysteine residues.

#### 3.3.2. 5-HT_1A_R

Among the large number of serotonin receptors, 5-HT_1A_ subtype has gained considerable attention for its role in the etiology and treatment of anxiety disorders. 5-HT_1A_R is coupled to various inhibitory G_i/o_ proteins, widely expressed in the nervous system and classified in two populations, based on their localization: presynaptic autoreceptors and postsynaptic heteroreceptors. The autoreceptors are distributed on 5-HT neurons in the raphe nuclei where they act as inhibitory feedback, negatively regulating 5-HT release, whereas heteroreceptors mediate 5-HT effects on mood, emotion and stress on target neurons expressed in the hippocampus, septum, amygdala and prefrontal cortex [105]. Alterations of 5-HT_1A_R expression or its pharmacological or genetic blockade lead to anxiety- and depression-like behaviors in animal models, while its overexpression reduces anxiety in mice [106]. In humans, mood disorders such as anxiety and depression have been associated with alteration in the expression pattern of 5-HT_1A_R, with an upregulation of autoreceptors and a downregulation of heteroreceptors [106,107]. Russo et al. [108] reported that CBD is an orthosteric ligand of 5-HT_1A_R, being able to displace, in a concentration-dependent manner, the classical agonist [^3^H]8-OH-DPAT from human 5-HT_1A_R-expressing CHO cells membranes, with a displacement of 73% at 16 μM. CBD acts as an agonist at this receptor, since at the same concentration, it increases [^35^S]GTPγS binding and reduces forskolin (FSK)-stimulated cAMP, an effect counteracted by the specific 5-HT_1A_R antagonist NAN-190. Later, Rock et al. [109] evaluated in vitro the ability of CBD to activate 5-HT_1A_R expressed at physiological levels in rat brainstem membranes in a 1 nM-10 μM range. At concentrations up to 10 μM, CBD was not able to displace [^3^H]8-OH-DPAT from specific binding sites on rat brainstem membranes. Then, they compared the ability of CBD and 8-OH-DPAT to stimulate [^35^S]GTPγS binding to rat brainstem membranes in a concentration-related manner. While 8-OH-DPAT induced such stimulation, no response was observed for CBD at any used concentration. Then, it was investigated whether CBD could act as positive allosteric modulator of 8-OH-DPAT in this concentration range. Indeed, CBD enhanced the ability of this agonist to stimulate [^35^S]GTPγS binding to rat brainstem membranes, since 100nM of CBD produced an upward shift in the concentration response curve of 8-OH-DPAT with a significant increase in E_max_ but not in EC_50_. The potentiating effect of CBD on 8-OH-DPAT was also confirmed in vivo, since CBD and 8-OH-DPAT synergistically suppress, at subthreshold doses, the LiCl-induced conditioned gaping reactions in rats. The involvement of 5-HT_1A_R in the anxiolytic and antidepressant-like effects of CBD has been documented in diverse studies [110,111,112]. For example, Zanelati et al. [110] showed that CBD induces antidepressant-like effects comparable to imipramine, an effect blocked by the 5-HT_1A_R antagonist WAY100635. The synergistic effect of CBD at 5-HT_1A_R was also demonstrated by Sales et al. [113], who showed that ineffective doses of CBD and fluoxetine, a serotoninergic anti-depressant, resulted in a significant anti-depressant-like effect. Moreover, the pretreatment with PCPA, an inhibitor of serotonin synthesis, abolishes CBD-induced behavioral effects in the forced swimming test, indicating the involvement of serotonin in CBD action. These results strongly corroborate the positive allosteric activity of CBD at 5-HT_1A_R. Norris et al. [114] reported that using targeted microinfusions of CBD into the shell region of the mesolimbic nucleus accumbens (NASh), CBD blocks the formation of fear-related memory and decreases ventral tegmental area (VTA) dopaminergic neuronal frequency and bursting activity through a mechanism mediated by 5-HT_1A_R, since both effects are reversed by the selective 5-HT_1A_R antagonist NAD 299. Moreover, intra-NASh CBD induces significant increases in non-dopaminergic, presumptive VTA GABAergic neurons. It was demonstrated, by a functional contralateral disconnection procedure, that the ability of intra-NASh CBD to block the formation of conditioned freezing behaviors was dependent on intra-VTA GABAergic transmission. These findings disclosed a novel circuit in the mesolimbic system between nucleus accumbens (NAc) and VTA, responsible for the observed effects of CBD on associative fear memory formation.

#### 3.3.3. GPR3 and GPR6

GPR3 and GPR6 are constitutionally active orphan receptors coupled to G_s_ protein, phylogenetically related to cannabinoid receptors [115]. They are widely expressed in the CNS where they are involved in several physio-pathological processes. GPR3 has been reported to play a role in the modulation of behavioral responses to stress in animal models used to evaluate emotional disorders including anxiety, depression-like disorders, and aggressiveness, by altering monoamine levels in various brain regions [116]. GPR6 is involved in instrumental learning and has been proposed as a therapeutic target for PD [117] and schizophrenia [118]. Laun and coll. [115] found, by screening a panel of cannabinoids against these receptors by a β-arrestin2 recruitment assay, that CBD acts, in a concentration-dependent manner, as an inverse agonist at both receptors, with higher activity at GPR6 (EC_50_ 0.18 vs. 1.22 μM). Such activity could contribute to rationalize the molecular mechanisms underlying the neuroprotective effect of CBD in animal models of PD [23]. Later, the same authors showed that CBD exhibits biased activity for GPR6 toward the β-arrestin2 recruitment pathway, being unable to significantly affect GPR6-mediated cAMP accumulation [119].

#### 3.3.4. GPR55

GPR55 is considered a novel cannabinoid-like receptor due to its nanomolar affinity for many endo-, synthetic- and phyto-cannabinoids [120]. GPR55 couples with G_α13_ protein and its stimulation leads to the downstream signaling pathway activation of rhoA, cdc42 and rac1 [120]. GPR55 is widely expressed in brain [121] and it has been proposed as a potential target for the treatment of anxiety and depression since its activation alleviates anxiety-like symptoms in mice subjected to acute stress [122]. However, it is unlikely that the anxiolytic effects of CBD are GPR55-mediated, due to its antagonist profile at this receptor (IC_50_ of 445 nM against the GPR55 agonist CP55940) [120]. Conversely, Kaplan et al. [123] showed that the efficacy of CBD in reducing seizures in a mouse genetic model of DS is mediated, at least in part, by its antagonism at GPR55, since the observed increase of inhibitory interneuron excitability is mimicked by CID16020046, a selective GPR55 antagonist. Moreover, GPR55 blockade by antagonists prevents the effects of CBD, further corroborating the role of GPR55 in the efficacy of CBD in such a DS model. In particular, high doses of CBD protect against seizures, while low doses improve autistic-like social deficit behaviors in DS mice, effects lost at higher doses. Such biphasic effect is similar to that exhibited by clonazepam. This latter compound acts as a positive allosteric modulator of GABA_A_Rs specific for α2/α3 subunit-containing receptor at low doses, while at higher doses it binds α1 subunit-containing receptor, inducing opposite effects in social behavior [124]. Thus, it is possible to speculate that low-dose effects of CBD reflect its positive allosteric modulation at α2 subunit-containing GABA_A_Rs (see Section 3.1.2) while at higher doses it targets GPR55, which is effective in controlling seizures.

### 3.4. σ1R

**Figure 4:**
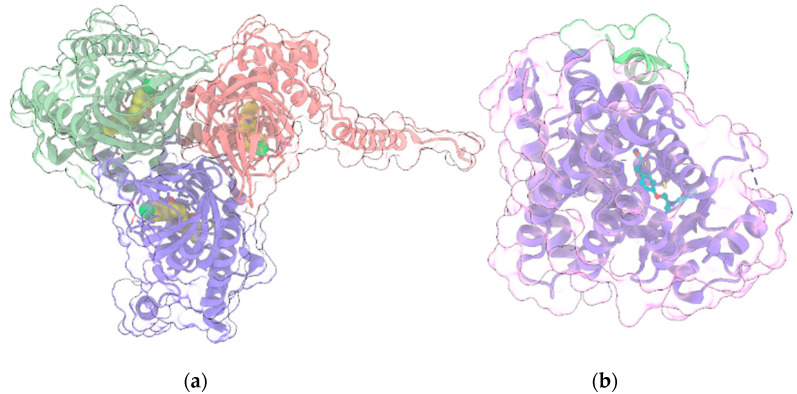
(**a**) Crystal structure (PDB entry: 6DJZ) of the 3:3 complex between σ1R (light green, red and violet ribbon and transparent surface for the three protein monomers) and the antagonist haloperidol (“sphere” representation with yellow C atoms); (**b**) Crystal structure (PDB entry: 5YCP) of the complex of PPARγ (light violet ribbon and transparent surface) with the agonist rosiglitazone (ball and stick with cyan C atoms) and the nuclear receptor coactivator 1 peptide (light green ribbon and transparent surface). In all panels, protein sidechains contacting ligands within 5 Å are shown as sticks. Oxygen, nitrogen, sulfur and chlorine atoms in ligands and visible protein sidechains are colored red, blue, yellow and green, respectively.

The human σ1R (Figure 4a) is a transmembrane regulatory protein involved in a wide range of physiological processes, including neurotransmission, calcium signaling and regulation of a large array of ion channels, G-protein coupled receptors and transcription factors.

Its crystallographic structure has been recently solved in complex with two ligands with different pharmacological profiles, i.e., the antagonist PD144418 and the agonist/inverse-agonist 4-IBP [125], unveiling a trimeric organization, with a single transmembrane helix for each protomer, located at each corner of the triangular trimer. The cytosolic domain of each protomer has a cupin-like β-barrel fold, which hosts a ligand in the central pocket, flanked by four α-helices. σ1R dysfunction has been implicated in a variety of neurological and neuropsychiatric disorders [126]. In particular, it has been shown [127] that σ1R, in tandem with the histidine triad nucleotide-binding protein 1 (HINT1), acts as on/off switch to control the association between GPCRs and NMDAR: σ1R agonists promote the interaction of GPCRs and NMDAR, whereas σ1R antagonists disrupt this association and prevent GPCRs from enhancing NMDAR function. Thus, σ1R antagonists represent a promising therapeutic strategy for the treatment of neuropsychiatric disorders where the NMDAR-mediated glutamatergic signaling plays a critical role [128]. Since some therapeutic indications for σ1R antagonists are common to those of CBD, Rodríguez-Muñoz and coll. [128] evaluated the effects of CBD in vitro and in animal models characterized by elevated activity of NMDAR, such as opioid analgesia attenuation, NMDA-induced convulsive syndrome and ischemic stroke. Indeed, in in vitro assay, CBD was shown to act similarly to a σ1R antagonist, disrupting the association of σ1R with the NR1 subunit of NMDAR, an effect prevented by σ1R agonists. Moreover, the in vivo positive effects of CBD on the aforementioned models are reduced by σ1R agonists and absent in σ1R^−/−^ mice. Collectively, these data suggest that CBD acts as σ1R antagonist by disrupting the association between NMDAR and GPCRs.

### 3.5. PPARγ

PPARγ (Figure 4b), along with the other two isoforms PPARα and PPARβ/δ, belongs to the group of Peroxisome proliferator-activated receptors (PPARs), a family of nuclear receptors [129] widely distributed in various organs and tissues. PPARs are ligand-activated transcription factors, which regulate the expression of their target genes upon heterodimerization with retinoid-X receptors (RXRs). Ligands bind to the ligand-binding pocket (LBP) of PPAR, inducing conformational changes that promote the recruitment of co-activators and the release of corepressors. Canonical agonists form a network of hydrogen bonds which stabilize the conformation of a short helix, the helix12 (H12), whereas partial agonists bind to distinct sub-regions or allosteric sites and activate these receptors through H12-independent mechanisms. Besides their well-consolidated role in glucose and lipid metabolism, PPARs also have anti-inflammatory and neuroprotective effects [130]. In particular, PPARγ is highly expressed in brain areas involved in the regulation of motivational and emotional behaviors [131]. Starting from the occurrence of PPARγ in neurons of the VTA, de Guglielmo et al. [132] demonstrated that PPARγ activation attenuates opioid consumption by reducing both the opioid motivation and its rewarding properties. These effects are associated with both a drastic reduction of heroin-induced elevation of the phosphorylation of DARPP-32 protein - a regulator of the efficacy of dopaminergic (DAergic) neurotransmission - in NAc, and a reduction of the acute heroin-induced NAc DA levels increase. Moreover, PPARγ activation attenuates the opioid-induced DA activity in the VTA. Domi et al. [131] investigated the role of PPARγ in anxiety and stress response in mice, showing that its activation prevents the anxiogenic effect of acute stress, while its ablation or the administration of a specific antagonist exacerbates basal anxiety and enhances sensitivity to stress. These results, combined with other studies including preclinical and clinical trials [133,134,135,136,137], demonstrate the therapeutic potential of PPARγ agonists for various neuropsychiatric disorders and drug dependencies. Interestingly, since CBD is a selective PPARγ agonist [19,138], many of its therapeutic effects in alleviating neuropsychiatric disorders could be mediated by PPARγ signaling and by its regulation of the mesolimbic DA activity [139]. Indeed, an increasing amount of evidence demonstrated that CBD modulates the mesolimbic DA system and that it represents a promising compound for the treatment of schizophrenia, due to its antipsychotic effects [140]. However, the modulation of VTA DA activity by CBD could also involve multiple mechanisms, including 5HT_1A_ activation [114] (see above).

## 4. Conclusions

**Figure 5:**
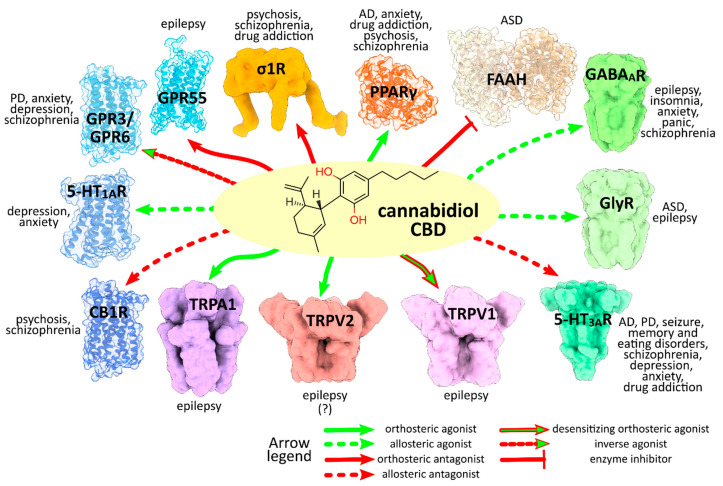
Activity pattern of CBD on target proteins related to neuropsychiatric disorders. The different regulatory activities exhibited by CBD are indicated by colored arrows as explained in the legend. The main neuropsychiatric disorders associated to each target are also reported. Abbreviations: Alzheimer’s Disease (AD), Parkinson’s Disease (PD), Autism Spectrum Disorders (ASD).

While the lack of selectivity/specificity for a given molecular target usually hampers or limits the pharmaceutical development of bioactive molecules, being associated with the occurrence of off-targets and undesirable side effects, an exception is made when the overall biological effect of a multitarget ligand results in an additive and consistent pharmacological profile (polypharmacology). Indeed, the risk associated with the reductionist approach ‘one-target-one-disease’ is to underestimate the biological pathway complexity entailed in human diseases where the physio-pathological processes are the result of a cross-talk among many interacting (macro)molecules. This is particularly relevant in the case of multifactorial, complex disorders related to the neuropsychiatric sphere. In this view, CBD represents a paradigmatic example of a polypharmacological agent able to synergistically modulate different targets involved in common signaling pathways/circuits within the nervous system, whose dysfunctions underlie many neurological and neuropsychiatric disorders (Figure 5).

For example, the restoration of NMDAR functionality by CBD could be due to both its negative allosteric modulation at CB1R and its σ1R antagonism. Moreover, the effect of CBD on the mesolimbic dopamine activity could arise by the stimulation of both 5-HT_1A_R and PPARγ. Yet, the elevation of inhibitory tone in CNS mediated by CBD occurs through its positive allosteric modulation of both GABA and glycine receptors. Indeed, CBD structural features, along with is its hydrophobic profile, make this compound particularly suitable to interact with protein binding sites embedded in membrane environments such as the allosteric/orthosteric binding sites of receptor channels and GPCRs. In this view, albeit a complex pharmacological profile has already emerged and it has been characterized to a good extent, other studies are necessary to completely decipher its mechanism of action and to disclose new potential pharmacological targets for this phytocannabinoid.

## Abbreviations

2-AG	2-arachidonoyl glycerol
5-HT	5-hydroxytryptamine/serotonin
5-HT*_n_*Rs	5-hydroxytryptamine subtype *n* receptors (*n* = 1A, 3)
8-OH-DPAT	8-hydroxy-2-(di-n-propylamino)tetralin
AD	Alzheimer’s disease
AEA	N-arachidonoylethanolamine/anandamide
AEDs	antiepileptic drugs
ARDs	ankyrin repeat domains
ASD	Autism spectrum disorder
BDNF	brain-derived neurotrophic factor
BDZ	benzodiazepine
CaM-Kinase II	Ca^2+^/calmodulin-dependent protein kinase II
cAMP	cyclic adenosine monophosphate
CB1R	cannabinoid receptor 1
CBD	Cannabidiol
CBDV	cannabidivarin
CNS	central nervous system
DRE	drug-resistant epilepsy
DS	Dravet syndrome
DZP	diazepam
ECD	extracellular N-terminal domain
EMA	European Medicines Agency
FAAH	fatty acid amide hydrolase
FDA	US Food and Drug Administration
FSK	forskolin
GABA	γ-aminobutyric acid
GABA*_N_*Rs	GABA *N* receptors (*N* = A,B)
GlyRs	glycine receptors
GPCRs	G-protein coupled receptors
GTPγS	guanosine 5′-O-[γ-thio]triphosphate
HINT1	histidine triad nucleotide-binding protein 1
HMGB1	high mobility group box 1
IL-*n*	interleukin *n* (*n* = 1β, 6)
LGS	Lennox-Gastaut syndrome
LTD	long-term depression
NAM	negative allosteric modulator
NMDA	N-methyl-D-aspartate
NMDAR	NMDA receptor
NMR	nuclear magnetic resonance
PD	Parkinson’s disease
PKA	protein kinase A
PKC	protein kinase C
PPAR	Peroxisome proliferator-activated receptor
PTZ	pentylenetetrazol
TCA	trans-cinnamaldehyde
TLE	temporal lobe epilepsy
TMD	transmembrane domain
TNF	tumor necrosis factor
TRPA1	transient receptor potential ankyrin subtype 1 protein
TRPV*n*	transient receptor potential cation channel subfamily V member *n* (*n* = 1–6)
VSLD	voltage-sensor like domain
Δ^9^-THC	Δ^9^-tetrahydrocannabinol
